# The gene coding for PGC-1α modifies age at onset in Huntington's Disease

**DOI:** 10.1186/1750-1326-4-3

**Published:** 2009-01-08

**Authors:** Patrick Weydt, Selma M Soyal, Cinzia Gellera, Stefano DiDonato, Claus Weidinger, Hannes Oberkofler, G Bernhard Landwehrmeyer, Wolfgang Patsch

**Affiliations:** 1 Department of Neurology, University of Ulm (P.W.; G.B.L.), Ulm, Germany; 2 Department of Laboratory Medicine, Paracelsus Medical University and Universitätsklinikum Salzburg (S.M.S.; C.W.; H.O.; W.P.), Salzburg, Austria; 3 Division of Biochemistry and Genetics, Fondazione IRCCS – Instituto Neurologico, C. Besta (C.G.; S.D.), Milan, Italy

## Abstract

Huntington's disease (HD) is one of the most common autosomal dominant inherited, neurodegenerative disorders. It is characterized by progressive motor, emotional and cognitive dysfunction. In addition metabolic abnormalities such as wasting and altered energy expenditure are increasingly recognized as clinical hallmarks of the disease. HD is caused by an unstable CAG repeat expansion in the HD gene (HTT), localized on chromosome 4p16.3. The number of CAG repeats in the HD gene is the main predictor of disease-onset, but the remaining variation is strongly heritable. Transcriptional dysregulation, mitochondrial dysfunction and enhanced oxidative stress have been implicated in the pathogenesis. Recent studies suggest that PGC-1α, a transcriptional master regulator of mitochondrial biogenesis and metabolism, is defective in HD. A genome wide search for modifier genes of HD age-of-onset had suggested linkage at chromosomal region 4p16-4p15, near the locus of *PPARGC1A*, the gene coding for PGC-1α. We now present data of 2-loci *PPARGC1A *block 2 haplotypes, showing an effect upon age-at-onset in 447 unrelated HD patients after statistical consideration of CAG repeat lengths in both HTT alleles. Block 1 haplotypes were not associated with the age-at-onset. Homozygosity for the 'protective' block 2 haplotype was associated with a significant delay in disease onset. To our knowledge this is the first study to show clinically relevant effects of the PGC-1α system on the course of Huntington's disease in humans.

## Background

Huntington's disease (HD [MIM 143100]; ) is one of the most common autosomal-dominant inherited neurodegenerative disorders. Clinically HD is characterized by motor and cognitive impairment, accompanied by a variable degree of personality change and psychiatric illness[1]. Advanced stages of HD are characterized by severe emaciation, despite a strong appetite and increased caloric intake[2,3]. HD is relentlessly progressive and patients succumb to the disease typically 10–25 years after disease onset[1]. In 1993, a CAG trinucleotide repeat expansion encoding an elongated polyglutamine tract in the huntingtin (HTT) protein was found to cause HD[4]. The number of CAG repeats in the *htt *gene is the most important, but not the only determinant of age at onset of HD. Depending on the populations studied, the number of CAG repeats in *htt *accounts for up to 73% of the variance in age at onset[5]. The remaining variation is strongly heritable[6]. Hence, modifier genes must contribute to the variability in age at onset of HD. The genetic modifiers identified so far include the huntingtin associated protein 1 (HAP1) gene and the ubiquitin carboxy-terminal hydrolase L1 (UCHL1) gene [7-9]. The MAPS study, a genome-wide scan for modifier genes of age at onset using micro-satellite markers at a 10-cM density, suggested linkage at chromosomes 4p16, 6p21-23 and 6q24-26 and more marginal associations at several other sites, including 4p15 (marker D4S3403)[10].

Recently, two independent groups presented evidence that the transcriptional co-regulator peroxisome proliferator-activated receptor γ (PPARγ) coactiavtor 1α (PGC-1α) plays a role in the neurodegeneration of HD [11-13]. PGC-1α regulates the expression of mitochondrial OXPHOS genes and endogenous antioxidants[14,15]. Mutant but not wild-type HTT down regulates the expression of this gene set[11,12]. Lack of PGC-1α expression produces a HD-like phenotype in mice and over-expression of PGC-1α can antagonize mutant HTT toxicity *in vitro *and *in vivo *[11,12,16,17]. *PPARGC1A*, the gene encoding PGC-1α, is localized on chromosome 4p15.1-2, a region, in proximity to one of the HD modifier loci identified in the MAPS genome scan[18]. We therefore hypothesized that *PPARGC1A *polymorphisms are associated with the age at onset in HD patients.

## Methods

### Clinical resource

We ascertained possible associations of age at onset with *PPARGC1A *in an Italian cohort of 449 HD patients. The age at onset was considered as the time when motor signs diagnostic of HD were first noted. All patients have been diagnosed genetically by the same laboratory at the Istituto Neurologico C. Besta of Milano[19]. As to the clinical evaluation, 139 patients were neurologically assessed at the Outpatients Neurogenetic Clinic facility of the C. Besta Institute (either by Stefano Di Donato, MD, and Caterina Mariotti, MD, Unit of Biochemistry and Genetics, or Paola Soliveri, MD, Division of Movement Disorders, Istituto Neurologico C. Besta, Milano). The remaining 310 patients were clinically assessed by expert neurologists from Italian Neurological Centers other than the Istituto Neurologico C. Besta, and then referred to us for molecular diagnosis. As age at onset in HD is difficult to ascertain, and susceptible to considerable variation on the basis of environmental and genetic factors[20], we set the alleged age at onset as the time when *motor signs diagnostic of HD *were first noted. With regard to patients referred from other neurological centres, we (SD and CG) carefully re-checked each file for age at onset. For most patients the presumed motor onset was clearly indicated; for a minority of patients, however, we found that the neurological onset came out to be different from the one suggested by the referring neurologist (possibly indicating the behavioural-psychiatric onset), and accordingly reset the disease onset as the age at motor onset.

The population comprised 215 male and 234 female unrelated HD patients. The mean (SD, median, range) of age at onset was 48.9 (13.9, 49, 6–80) years. The mean (SD, median, range) of HD CAG repeat size as determined in a single diagnostic laboratory was 45.3 (5.5, 44, 37–90). HD CAG repeat size explained 61% of the variation in age at onset and no sex-specific difference in age at onset or HD CAG repeat size was observed.

### Genotyping

DNA was isolated from peripheral white blood cells. By sequencing phased chromosomes and typing eight informative single nucleotide polymorphisms (SNPs) of *PPARGC1A *in various populations, we previously identified two haplotype blocks, termed block 1 and 2, each comprising five common haplotypes. The boundary between the two haplotype blocks is located in intron 2. Haplotype block 1 extends 20 kb upstream of the translational start site, while haplotype block 2 extends < 20 kb beyond the proximal poly A signal[21]. Four SNPs discriminatory for *PPARGC1A *haplotype block 1 at gene positions -3974 A/G (rs2970865), -3833 A/C (rs1878949), -1694 T/C (rs17576121), and -1437A/G (rs2970870) as well as four haplotype block 2 SNPs at gene positions +75657 C/T (rs2970847), +75919 C/T (rs8192678), +76059 C/T (rs3755863) and 94581 C/T (rs6821591) were typed in 389 HD patients. SNPs rs2970847, rs8192678, rs3755863 and rs6821591 in the coding region correspond to positions +1302, +1564, +1704 and +2962, respectively, in the mRNA sequence relative to the translational start site. Among variant sites, only rs8192678 results in an amino acid change (Gly > Ser). SNP qualifiers refer to database entries . *PPARGC1A *haplotype block 1 SNPs were determined using TaqMan Genotyping Assays (Applied Biosystems, Warrington, UK) C_1643250_10, C__1643249_10, C_27842167_10 and C_1643241_10. TaqMan Assays for haplotype block 2 SNPs were PGC1ASNPEI_301 (custom), C_1643192_10, C_25992571_10 and C_26497328_10, respectively. The overall genotyping success rate was 99%. Success rates for all SNPs typed were > 99% with the exception of rs1878949 which was 96%. In several subjects in whom typing of rs1878949 was unsuccessful, the presence of the region harboring the SNP was verified by sequencing to exclude major sequence deviations. Correct typing results were verified in > 15% of subjects by restriction enzyme digestion and/or sequencing.

### Statistics

Associations of SNPs with age at onset of HD were ascertained in linear models. Logarithmically transformed age at onset was used as the dependent variable and individual SNPs, normal and expanded CAG repeat sizes as well as their interactions as independent variables[22]. For testing associations between haplotypes and age at onset, we used the haplo.score software, which provides both global and haplotype-specific tests[23]. Adjustments were made for normal CAG repeat, expanded CAG repeat size and their interaction. The THESIAS software  was used to estimate standardized pairwise linkage disequilibria (LD) expressed in terms of D', haplotype frequencies and covariate-adjusted mean effects of haplotypes on logarithmically transformed age at onset.

## Results

**Figure 1:**
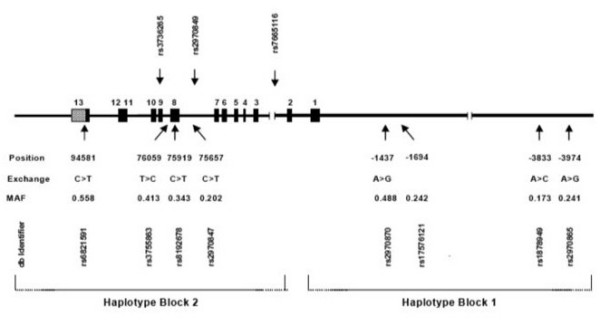
**Polymorphisms and haplotype blocks in PPARGC1A**. Linear map with exons (full boxes), SNP positions are relative to the translational start site. MAF indicates minor allele frequency; typing studies in other populations showed that, unlike in the HD population studied, C > T at rs6821591; SNP qualifiers refer to database entries . SNPs not used in the initial haplotyping studies are shown above the linear map. The extension of haplotype blocks is shown at the bottom. Scales differ for the transcribed sequence and the 5'-untranscribed sequence.

Distributions of genotypes at all SNPs did not deviate significantly from Hardy-Weinberg expectations. As expected, the pairwise LD matrix revealed two main haplotype blocks, previously identified in other populations[21]. In each haplotype block, 5 common haplotypes with frequencies > 0.01 were inferred that accounted for > 97% of the chromosomes. For each of the common haplotypes, the squared correlation between true and predicted haplotype dose was > 0.97 (Fig. 1). No associations were observed between block 1 haplotypes and age-at-onset (data not shown). Rs6821591, located in haploblock 2 in the 3-untranslated region, displayed an association in the dominant model (*P *= 0.0178). Furthermore, global testing suggested an association between block 2 haplotypes and age at onset (Table 1). In particular, haplotype-specific statistics scores were highest and lowest for haplotypes 0001 and 0000, respectively. The estimated difference in age at onset between these two haplotypes was 2.8 years. Consequently, rs6821591, discriminating haplotypes 0001 and 0000, was found to be associated with the age at onset after adjustment for linkage disequilibrium between the SNPs forming haploblock 2 (*P *= 0.0025).

**Table 1:** *PPARGC1A *Block 2 Haplotypes and Age at Onset of HD

Haplotype	Frequency	Score	*P*
0000	0.234	-2.766	0.0057
0001	0.139	2.633	0.0085
0011	0.080	0.038	0.9698
0111	0.324	-0.192	0.8479
1000	0.204	0.396	0.6923

N = 389; adjusted for CAG repeat size on both alleles (HD and non-HD) and their interaction;

*P *= 0.0161 for global haplotype statistics; block 2 haplotypes: 0, more common allele

(+1302C, +1564C, +1704C, +2962C); 1, less common allele (+1302T, +1564T, +1704C, +2962T).

To identify individual SNPs in this region that may show stronger associations with age at onset, we searched HapMap, release 21a/phase II  for SNPs with variant alleles predominantly occurring on block 2 haplotypes 000X. We identified several SNPs and typed three such SNPs (rs2970849, rs25935762, rs31179675) in the 389 HD patients. According to phased HapMap data, these three SNPS signify distinct clades of 000X block 2 haplotypes. rs2970849 (C/T), located in intron 7, and rs3736265 (A/G, Thr/Met), located in exon 9, showed no associations with age at onset, irrespective of the model used (data not shown). However, rs7665116 (A/G), located at the 3'-end of a highly conserved region in intron 2, revealed a significant association in the additive and dominant model (both *P *< 0.002). We therefore typed rs7665116 in the 60 remaining HD patients. Considering CAG repeat size in both alleles as well as their interaction in the 449 study subjects, a significant association of rs7665116 with age at onset was observed in both the additive and the dominant model, and the age of onset increased by 3.7 or 4 years in going from the A/A to the A/G or G/G genotypes, respectively (Table 2). The G/G genotype, which showed the biggest difference in age at onset, was present in 12 cases. The statistical significance of the association was maintained after the Bonferroni correction for the number of SNPs tested (*P *< 0.02 and *P *< 0.005 for additive and dominant models, respectively). Introducing rs7665116 explained 2.6% of the residual variance in the model. No interaction of rs7665116 with HD CAG repeat size was noted.

**Table 2:** *PPARGC1A *rs7665116 and Age at Onset in the HD Cohort

	rs7665116 genotype				
					
Variable	A/A	A/G	G/G	*P*^a^	*P*^b^
Sex, m/f	155/157	53/72	7/5	n.s.	n.s.
HD CAG	45.3 (5.5)	45.2 (5.9)	45.8 (2.8)	n.s.	n.s.
Non-HD CAG	18.3 (3.2)	18.5 (3.5)	17.7 (3.9)	n.s.	n.s.
HD CAG*non-HD CAG	828 (179)	840 (210)	809 (192)	n.s.	n.s.
HD-onset, years^c^	45.08 (1.43)	48.75 (1.39)	49.09 (1.22)	0.0016	0.0003

^a ^additive model; ^b ^dominant model; ^c ^calculated from log-transformed years and adjusted for HD CAG and non-HD CAG repeat size and their product.

The linkage disequilibrium between rs7665116 and rs6821591 was not complete (D' = -0.86). R^2 ^was 0.098, reflecting the lower frequency of rs7665116. We therefore typed rs6821591 in the remaining subjects and confirmed the associations observed in the smaller number of HD patients (Table 3). We also ascertained associations of two-loci haplotypes and, as expected, found opposing associations of haplotypes carrying two wild-type and two variant nucleotides (Table 4). The estimated difference in age-at-onset was 2.1 years.

**Table 3:** *PPARGC1A *rs6821591 and Age at Onset in the HD Cohort

	Rs6821591 genotype				
					
Variable	A/A	A/G	G/G	*P*^a^	*P*^b^
Sex, m/f	39/41	110/121	64/72	n.s.	n.s.
HD CAG	45.2 (5.5)	44.8 (5.9)	46.1 (2.8)	n.s.	n.s.
Non-HD CAG	18.5 (5.4)	18.2 (3.9)	18.5 (7.5)	n.s.	n.s.
HD CAG*non-HD CAG	833 (158)	816 (170)	855 (228)	n.s.	n.s.
HD-onset, years^c^	44.1 (1.48)	46.6 (1.34)	46.9 (1.48)	0.0870	0.0295

^a ^additive model; ^b ^dominant model; ^c ^calculated from log-transformed years and adjusted for HD CAG and non-HD CAG repeat size and their product.

**Table 4:** PPARGC1A rs7665116 and rs6821591 Haplotypes and Age-at-Onset of HD

Haplotype	Frequency	Score	*P*
00	0.427	-2.065	0.0389
01	0.406	-0.583	0.5600
10	0.010	0.812	0.4170
11	0.157	3.404	0.0007

N = 447; adjusted for CAG repeat size on both alleles (HD and non-HD) and their interaction;

*P *= 0.0056 for global haplotype statistics; block 2 haplotypes: 0, more common allele

(rs7665116A, rs6821591C); 1, less common allele (rs7665116G, rs6821591T).

## Discussion

Here we report the presence of a common polymorphism and a common haplotype in *PPARGC1A *that are associated with a delay in age at onset of motor symptoms in patients with Huntington's disease. This clinical finding complements independent mechanistic studies on transgenic animals and human *post mortem *brain tissue, which demonstrated that impairment of the PGC-1α system contributes to the pathology of experimental HD. Lin et al. showed that mutant htt suppresses the expression of PGC-1α, while Weydt et al. found that mutant htt can inhibit the effects of PGC-1α on the expression of its target genes [12,16]. These two concepts are not mutually exclusive, as PGC-1α may induce its own expression via a feed-forward loop[15,24]. Our association study in humans now suggests that *PPARGC1A *indeed modifies the age at onset of HD and hence provide critical support for a role of PGC-1α in the pathogenesis of HD in humans.

It should be noted, that clinical phenotypes reminiscent of HD have been described without mutations in the HD gene[25]. Interestingly, chromosomal region 4p15.3 has been implicated in a recessive, progressive neurodegenerative Huntington-like disorder[26] (HDL3 [MIM 604802]; ). It is thus possible that "loss of function" mutations in *PPARGC1A *can cause a recessive HD-like disease as suggested by gene deletion studies in mice[16,17].

The arguments presented here provide first support from observations in humans for the concept that PGC-1α failure contributes to the pathogenesis of HD. If our results are confirmed in other populations, the identification of the functional SNP(s) may provide mechanistic insight into the pathogenesis of HD and may have important implications for the delineation of therapeutic targets[27,28].

## Competing interests

The authors declare that they have no competing interests.

## Authors' contributions

PW conceived of the study, participated in its design and coordination and drafted the manuscript. SMS designed and carried out the molecular genetic investigation strategy. CG and SD contributed the clinical and biological sample sets and participated in the data analysis. CW and HO participated in the molecular genetic studies and the data analysis. GBL participated in the design of the study and the drafting of the manuscript. WP participated in the design of the study, performed the statistical analysis and coordinated and drafted the manuscript.
